# Supplementary material for: Activated Regulatory T-Cells, Dysfunctional and Senescent T-Cells Hinder the Immunity in Pancreatic Cancer
Source: Cancers (Basel). 2021 Apr 8;13(8):1776. doi: 10.3390/cancers13081776 (PMC8068251; doi:10.3390/cancers13081776)
Supplement: Supplementary file 1 [file cancers-13-01776-s001.zip › supplementary material/cancers-1145443-crosscheck-suppl-final.docx]

Supplementary Material: Activated Regulatory T-Cells,
Dysfunctional and Senescent T-Cells Hinder the Immunity in Pancreatic Cancer

Shivan Sivakumar , Enas Abu-Shah, David J. Ahern, Edward H. Arbe-Barnes, Ashwin K. Jainarayanan,
Nagina Mangal, Srikanth Reddy, Aniko Rendek, Alistair Easton, Elke Kurz, Michael Silva, Zahir Soonawalla,
Lara R. Heij, Rachael Bashford-Rogers, Mark R. Middleton and Michael L. Dustin

**Table S1.** Patient data. PanIn= Pancreatic intraepithelial neoplasm. IPMN= Intraductal papillary mucinous neoplasm.

| **Patient** | **Sex** | **Age** | **TNM Stage** | **Size of Primary** | **Background Pancreas Related Pathology** | **Stent before Surgery** | **Other Co-Morbidities** | **Baseline Ca19.9** |
| --- | --- | --- | --- | --- | --- | --- | --- | --- |
| 1 | Male | 71 | pT3 N2 (11/52) L1 V1 R1(Posterior, Medial) | 45 mm | PanIn | No | No | N/A |
| 2 | Male | 72 | pT2 N1 (3/36) L1 V1 R0 | 28 mm | PanIn | Yes | Diabetes mellitus, Acute kidney injury (Stage 1), Ex-smoker. | N/A |
| 3 | Female | 51 | pT2 N2 (12/66) M0 L1 V1 R2(Medial) | 26 mm | IPMN | No | Diabetes mellitus. | Ca19.9 - 18 IU/mL |
| 4 | Male | 80 | pT3 N0 (0/22 + 0/1 = 0/23)  M0 L0 V0 Pn1 R0 | 15 mm | No | No | Cancer of prostate. | Ca19.9 - 1356 U/mL |
| 5 | Female | 73 | R1 (medial × 2 & pancreatic RM) pT3 L1 V0 Pn1 N2 (16/37) | 42 mm | No | Yes | No | N/A |
| 6 | Female | 67 | pT3, N2 (10/68 M0, L1 V1 R1 anterior medial) | 28 mm | PanIn | No | Inactive: Ex-smoker | Ca19.9 - 147 U/mL |
| 7 | Male | 67 | R1 (posterior) pT2 L0 V1 Pn1 N1(1/19) | 24 mm | PanIn | No | Smoker. | N/A |
| 8 | Male | 69 | pT2 N2 (9/18) M0 L1 V1 R0 | 28 mm | PanIn | No | Diabetes mellitus, Ex-smoker. | N/A |

**Table S2.** CYTOF panel.

| **Antigen** | **Metal Tag** | **Clone** | **Vendor, Catalogue No.** |
| --- | --- | --- | --- |
| CD45 | 89 Y | HI30 | Fluidigm, 3089003B |
| CD14 Qdot | Qdot655 (114 Cd) | TuK4 | ThermoFisher, Q10056 |
| EpCAM | 141 Pr | 9C4 | Fluidigm, 3141006B |
| CD19 | 142 Nd | HIB19 | Fluidigm, 3142001B |
| CD127 | 143 Nd | A019D5 | Fluidigm, 3143012B |
| HLA-A,B,C | 144 Nd | W6/32 | Fluidigm, 3144017B |
| CD4 | 145 Nd | RPA-T4 | Fluidigm, 3145001B |
| CD8a | 146 Nd | RPA-T8 | Fluidigm, 3146001B |
| CD39 | 147 Sm | A1 | Biolegend, 328221 |
| ICOS | 148 Nd | C398.4A | Fluidigm, 3148019B |
| CD25 | 149 Sm | 2A3 | Fluidigm, 3149010B |
| OX40 | 150 Nd | ACT35 | Fluidigm, 3150023B |
| CD103 | 151 Eu | BerACT8 | Fluidigm, 3151011B |
| CD66b | 152 Sm | 80H3 | Fluidigm, 3152011B |
| Tigit | 153 Eu | MBSA43 | Fluidigm, 3153019B |
| Tim-3 | 154 Sm | F382E2 | Fluidigm, 3154010B |
| CD27 | 155 Gd | L128 | Fluidigm, 3155001B |
| PD-L1 | 156 Gd | 29E.2A3 | Fluidigm, 3156026B |
| CD33 | 158 Gd | WM53 | Fluidigm, 3158001B |
| GITR | 159 Tb | 621 | Fluidigm, 3159020B |
| CD28 | 160 Gd | CD28.2 | Fluidigm, 3160003B |
| CTLA-4 | 161 Dy | 14D3 | Fluidigm, 3161004B |
| FoxP3 | 162 Dy | PCH101 | Fluidigm, 3162011A |
| CD56 | 163 Dy | NCAM16.2 | Fluidigm, 3163007B |
| CD15 | 164 Dy | W6D3 | Fluidigm, 3164001B |
| Lag-3 | 165 Ho | 11C3C65 | Fluidigm, 3165037B |
| CCR7 | 167 Er | G043H7 | Fluidigm, 3167009A |
| CD40L | 168 Er | 2431 | Fluidigm, 3168006B |
| CD45RA | 169 Tm | HI100 | Fluidigm, 3169008B |
| CD3 | 170 Er | UCHT1 | Fluidigm, 3170001B |
| Granzyme B | 171 Yb | GB11 | Fluidigm, 3171002B |
| Ki67 | 172 Yb | B56 | Fluidigm, 3172024B |
| 4-1BB | 173 Yb | 4B4-1 | Fluidigm, 3173015B |
| HLADR | 174 Yb | L243 | Fluidigm, 3174001B |
| PD-1 | 175 Lu | EH12.2H7 | Fluidigm, 3174020B |
| CD57 | 176 Yb | HCD57 | Fluidigm, 3176019B |
| CD16 | 209 Bi | 3G8 | Fluidigm, 3209003B |

**Table S3.** Cell abundance (Figure 1B).

| **Patient** | **%CD45+EpCAM− (Immune)** | **%CD45-EpCAM+ (Epithelium)** | **%CD45-EpCAM− (Stroma)** | **%CD45+EpCAM+ (EpCAM+ Immune Cells)** |
| --- | --- | --- | --- | --- |
| PT1 | 16.5898 | 26.485 | 56.5419 | 0.3832 |
| PT2 | 35.7895 | 19.4272 | 44.2292 | 0.5542 |
| PT3 | 14.2615 | 37.1795 | 47.6864 | 0.8727 |
| PT4 | 5.5447 | 16.5553 | 77.6596 | 0.2404 |
| PT5 | 45.5499 | 18.8121 | 31.9863 | 3.6517 |
| PT6 | 24.1776 | 39.1017 | 33.9784 | 2.7424 |
| PT7 | 19.3847 | 30.4133 | 49.6076 | 0.5943 |
| PT8 | 21.5233 | 18.504 | 58.7762 | 1.1965 |

**Figure S1**. Individual patients’ immune populations. (**A**) Expression of clustering markers on top of a FlowSOM tree showing the relationship between metaclusters, for the pooled single cell data. Population frequency is presented in the pie chart in each circle along the tree, the background colour represents the metacluster identity. Metaclusters in these trees have higher granularity to allow for identifying additional features in the data compared to the summary in main figure the corresponding between here and main figure are: MC10 *(*in Figure. 1D) = MC10 + 12 (in Figure S1*)*. (**B**) FlowSOM trees showing the individual metacluster frequency for each patient. The size of the pie chart corresponds to the frequency relative frequency. (Expression profile: from low (Dark blue) to high (Deep red)).


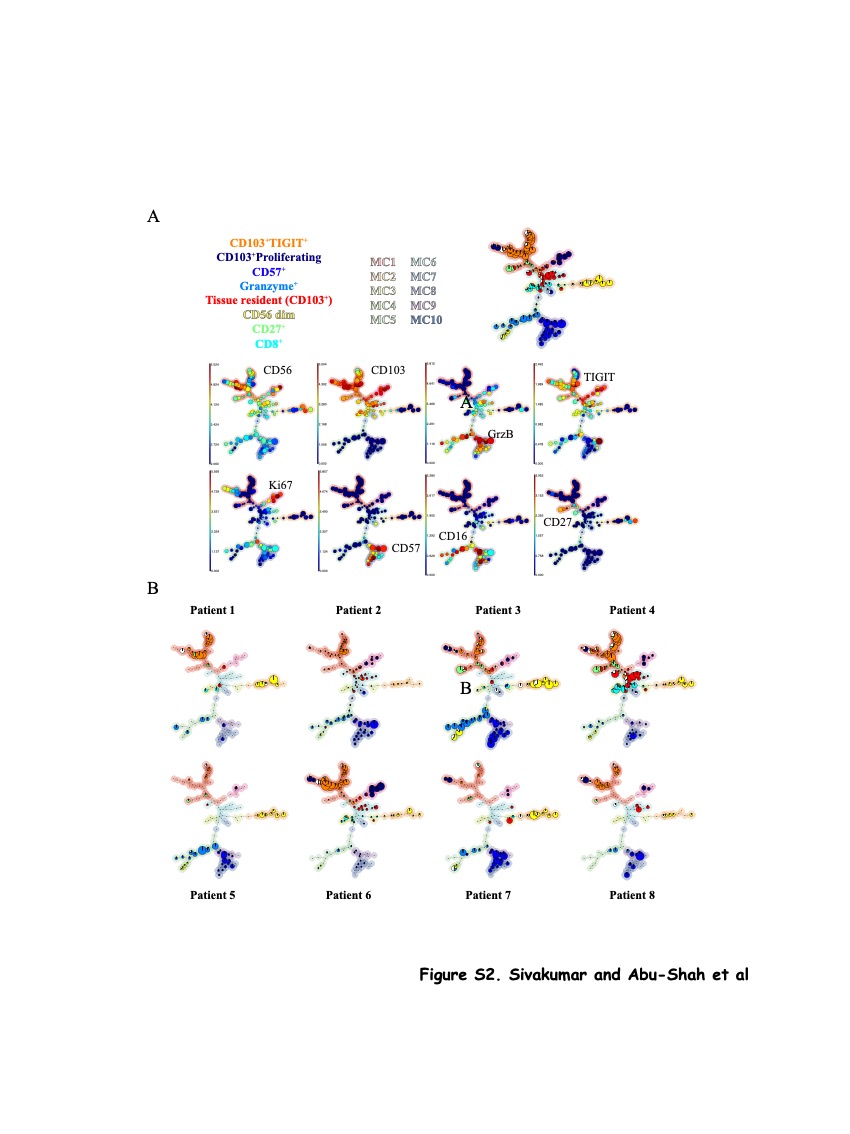


**Figure S2.** Individual patients’ NK populations. (**A**) Expression of clustering markers on top of a FlowSOM tree showing the relationship between metacluster, for the pooled single-cell data (**A**; ~2500 cells). Population frequency is presented in the pie chart in each circle along the tree, the background colour represents the metacluster identity. Metaclusters in these trees have higher granularity to allow for identifying additional features in the data compared to the summary in main Figure 2. (in Figure. 1E) = MC2 + 4 (in Figure S2), MC6 = MC7 + 8 + 9. (**B**) FlowSOM trees showing the individual metacluster frequency for each patient. The size of the pie chart corresponds to the frequency relative frequency. (Expression profile: from low (Dark blue) to high (Deep red)).


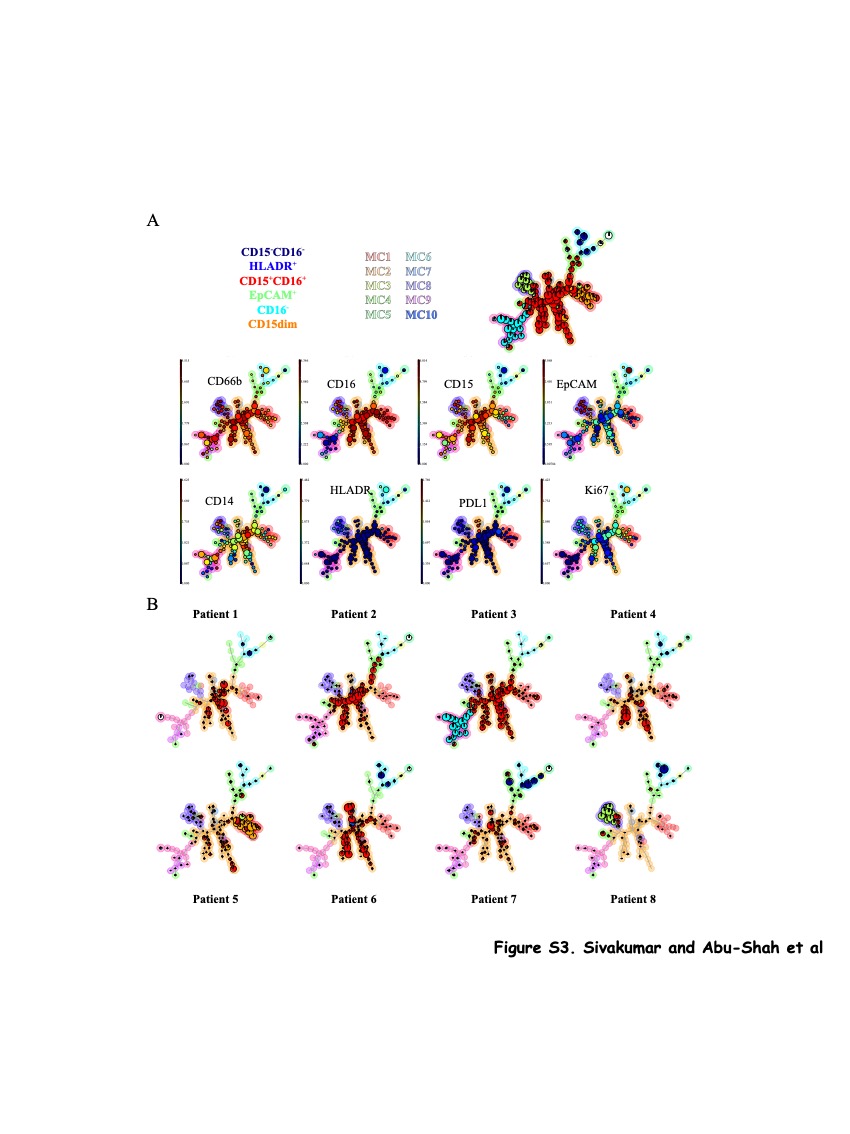


**Figure S3.** Individual patients’ Granulocyte populations. (**A**) Expression of clustering markers on top of a FlowSOM tree showing the relationship between metacluster, for the pooled single-cell data (**A**; ~36,000 cells). Population frequency is presented in the pie chart in each circle along the tree, the background colour represents the metacluster identity. Metaclusters in these trees have higher granularity to allow for identifying additional features in the data compared to the summary in main figure the corresponding between here and main figure are: MC3 (in Figure 1F) = MC3+4+6 (in Figure S3). (**B**) FlowSOM trees showing the individual metacluster frequency for each patient. The size of the pie chart corresponds to the frequency relative frequency. (Expression profile: from low (Dark blue) to high (Deep red)).


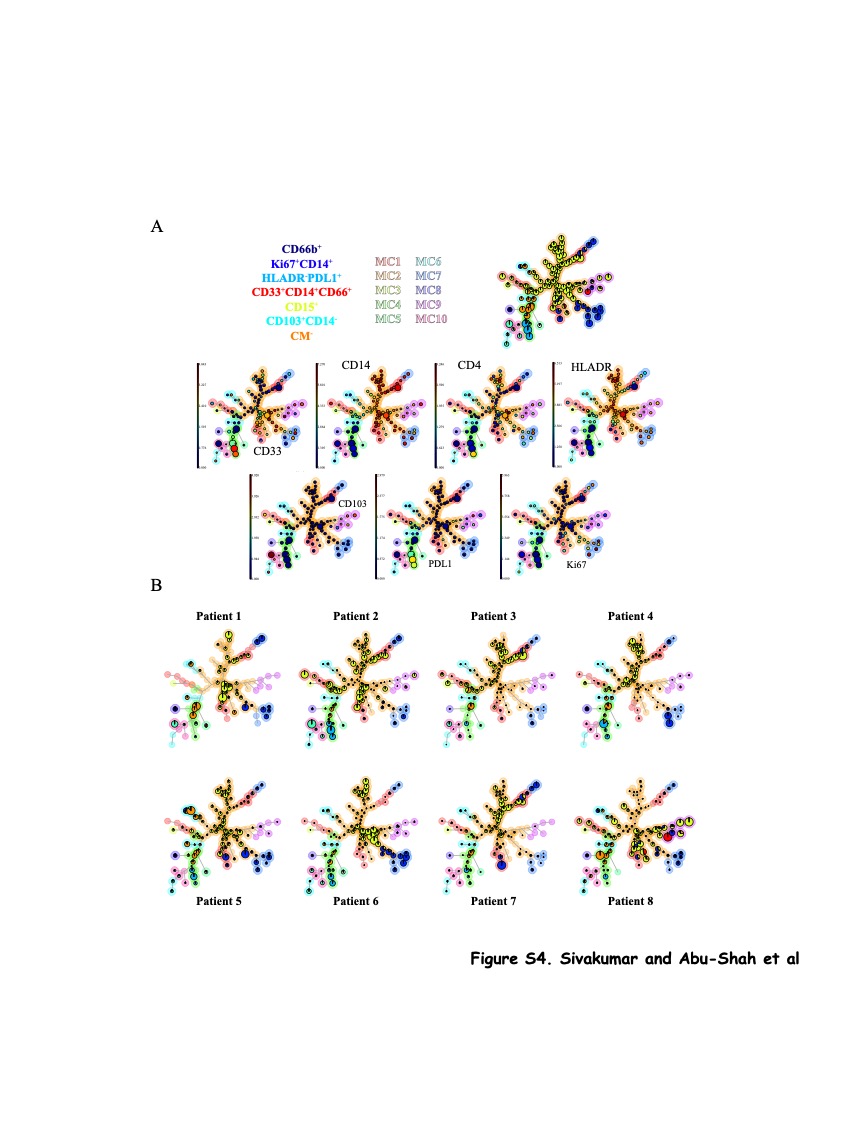


**Figure S4.** Individual patients’ Mononuclear Phagocytes populations. (**A**) Expression of clustering markers on top of a FlowSOM tree showing the relationship between metacluster, for the pooled single-cell data (**A**; ~20,260 cells). Population frequency is presented in the pie chart in each circle along the tree, the background colour represents the metacluster identity. Metaclusters in these trees have higher granularity to allow for identifying additional features in the data compared to the summary in main figure the corresponding between here and main figure are: MC2 (in Figure 1G) = MC2 + 9 (in figure S4); MC3 = MC3 + 4; MC4 = MC5 + 6. (**B**) FlowSOM trees showing the individual metacluster frequency for each patient. The size of the pie chart corresponds to the frequency relative frequency. (Expression profile: from low (Dark blue) to high (Deep red)).


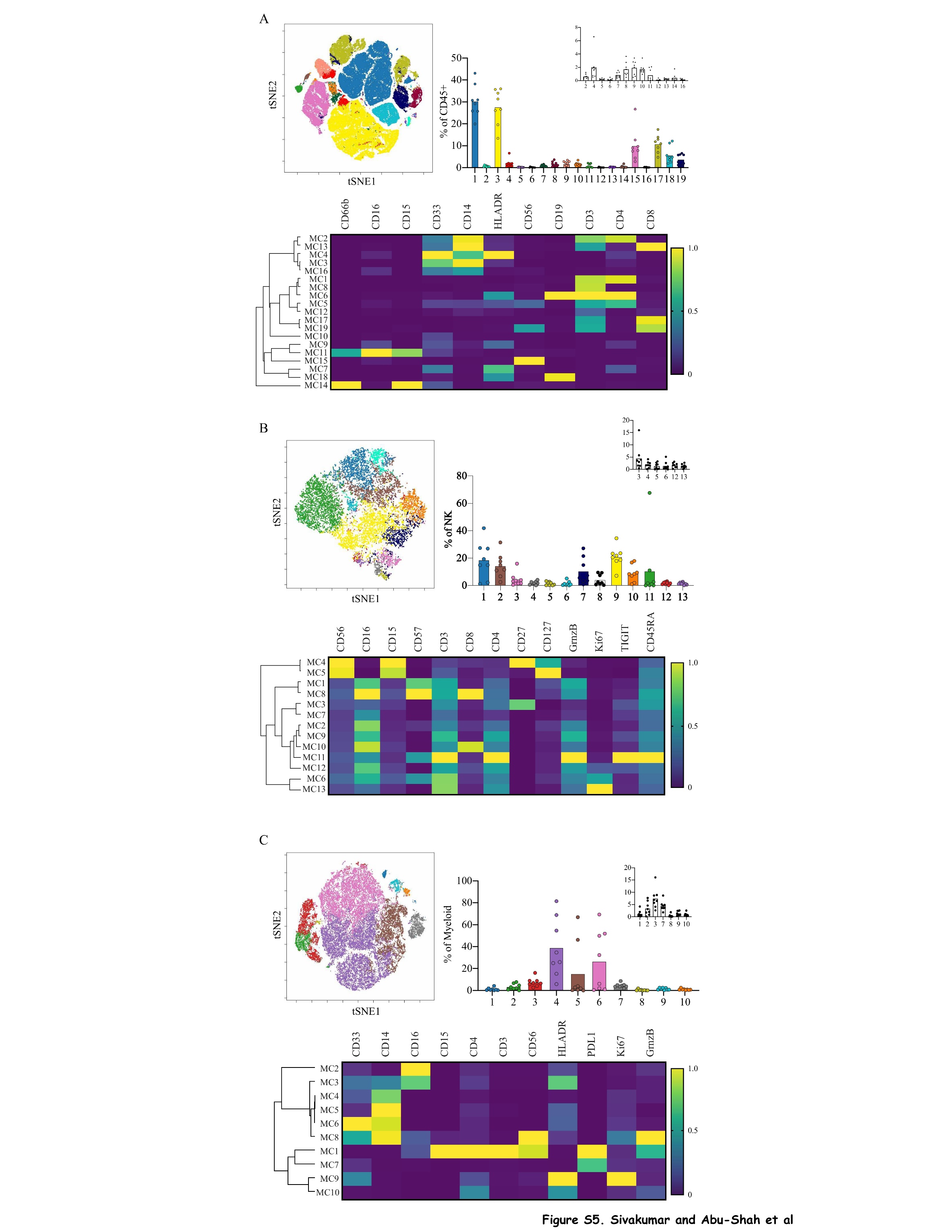


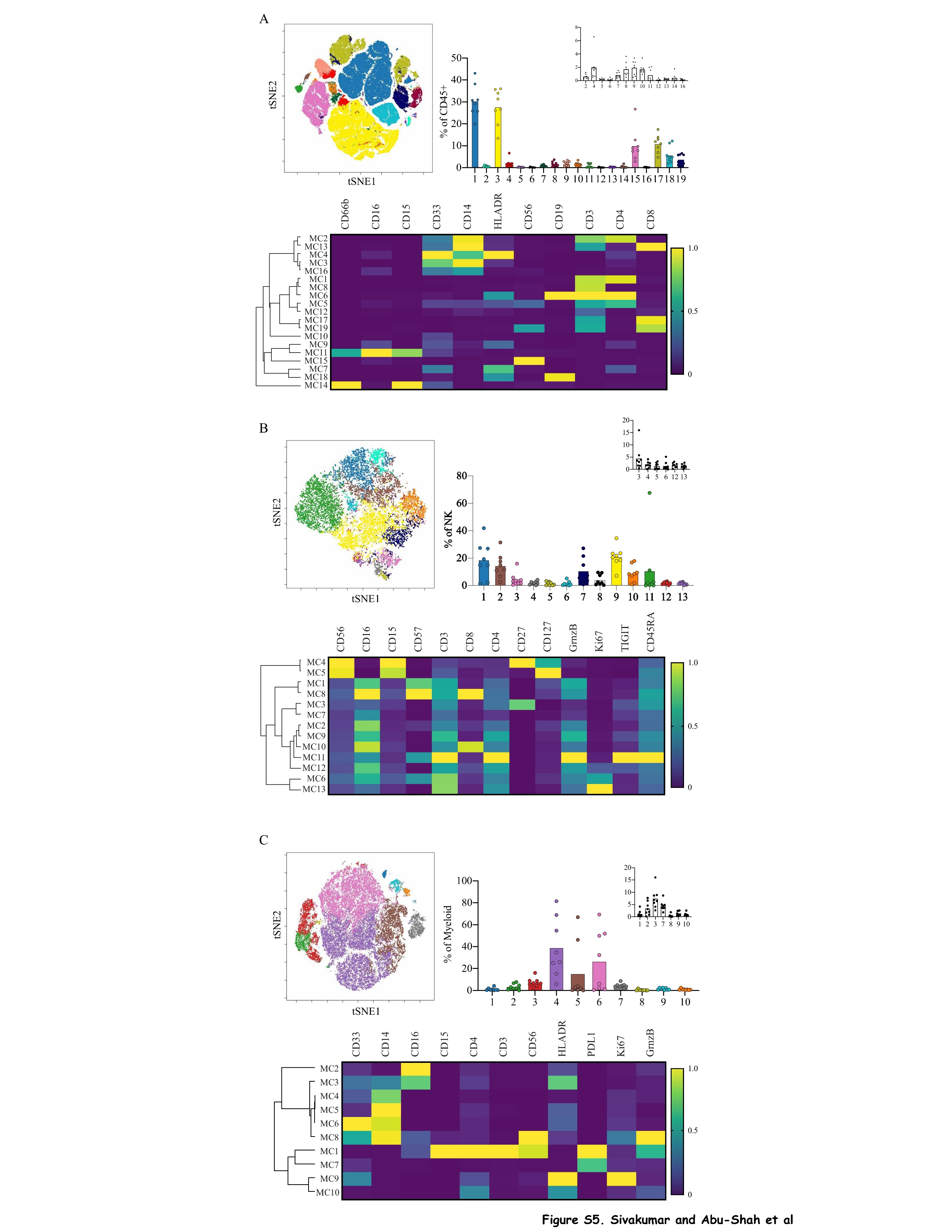


**Figure S5.** Immune-complexes and MDSC observed in peripheral blood of PDAC patients. (**A**) 124,000 CD45+ cells pooled from 8 patients and viSNE analysis using main cell lineage markers was performed to identify the main immune cell populations. The main immune populations coloured and labelled by FlowSOM. Bar plots of metacluster frequencies in each patient. Inset shows the lower frequency metaclusters. Heatmap of FlowSOM metaclusters of CD45+ cells; rows represent metaclusters from combined single cells across patients. CD66+ represent low density neutrophils and CD14+CD3+ are T-cell/monocyte complexes. (**B**) ~12,750 NK cells were pooled and 13 different metaclusters identified with FlowSOM. Bar plots show metacluster frequencies. Inset shows the lower frequency metaclusters. Expression profile is presented in the heatmap. Most metacluster are Granzyme B+, indicative of cytolytic capacity. (**C**) ~42,700 Mononuclear Phagocyte cells were pooled and 13 different metaclusters identified with FlowSOM. Bar plots show metacluster frequencies. Inset shows the lower frequency metaclusters. Expression profile is presented in the heatmap. The main metacluster appear to be MDSCs. All bar plots are median and the individual dots are individual patients. Heatmaps are normalised for each marker.


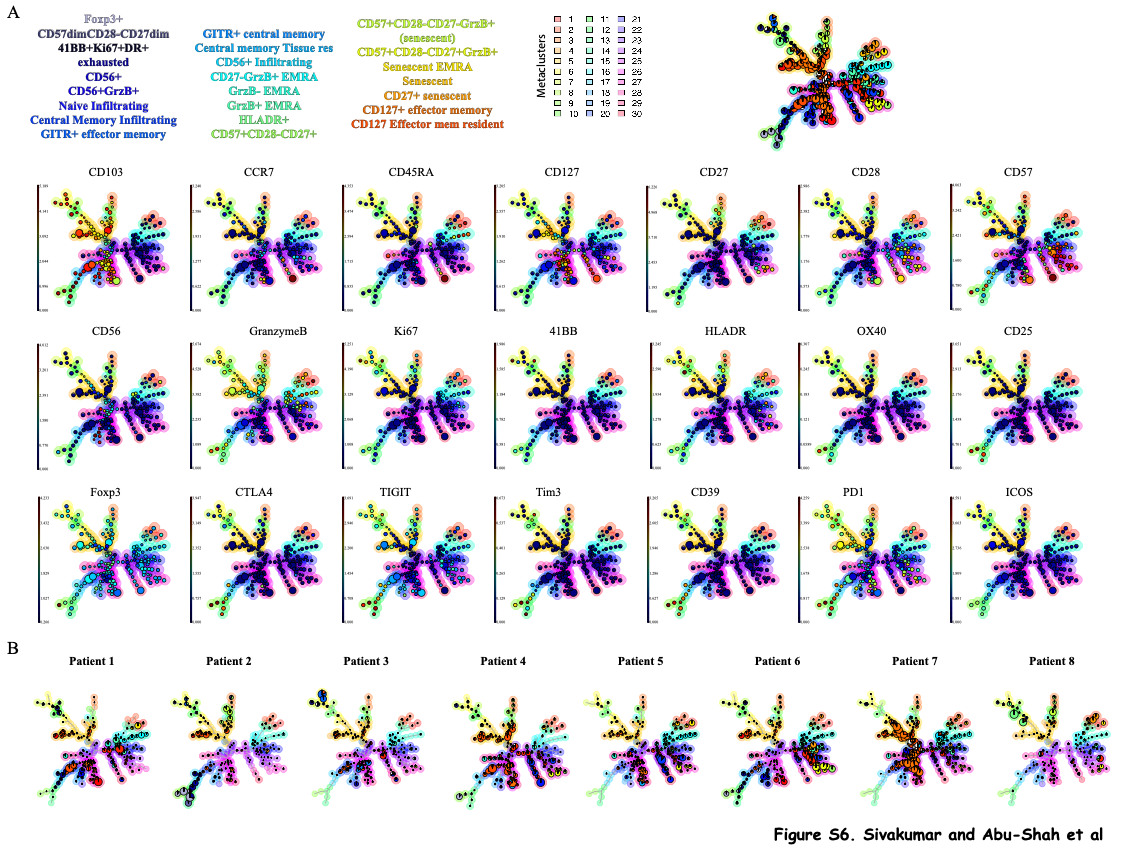


**Figure S6.** Individual patients’ CD8+ T-cells populations. (**A**) Expression of clustering markers on top of a FlowSOM tree showing the relationship between metacluster, for the pooled single-cell data (**A**). Population frequency is presented in the pie chart in each circle along the tree, the background colour represents the metacluster identity. Metaclusters in these trees have higher granularity to allow for identifying additional features in the data compared to the summary in main figure the corresponding between here and main figure are: MC1 (in Figure 2A) = MC4 + 21 + 24 + 25 + 27 + 28 + 29 (in Figure S6); MC2 = MC5 + 20 + 23 + 26; MC3 = MC18 + MC19; MC4 = MC2 + 3 + 14; MC6 = MC12 + 13 + 17; MC9 = MC7 + 8; MC10 = MC6 + MC11. (**B**) FlowSOM trees showing the individual metacluster frequency for each patient. The size of the pie chart corresponds to the frequency relative frequency. (Expression profile: from low (Dark blue) to high (Deep red)).


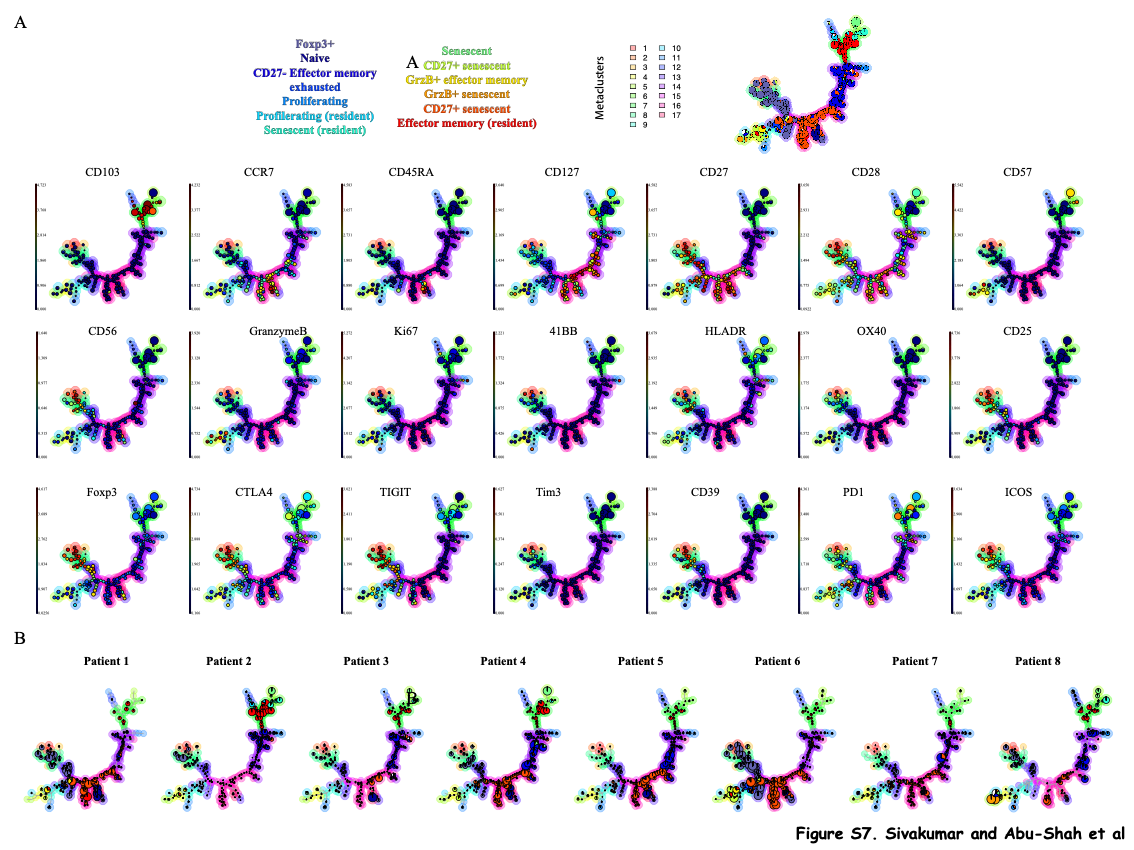


**Figure S7.** Individual patients’ CD4+ T-cells populations. (**A**) Expression of clustering markers on top of a FlowSOM tree showing the relationship between metacluster, for the pooled single-cell data (**top**). Population frequency is presented in the pie chart in each circle along the tree, the background colour represents the metacluster identity. Metaclusters in these trees have higher granularity to allow for identifying additional features in the data compared to the summary in main figure the corresponding between here and main figure are: MC1 (in Figure 2B) = MC1 + 2 + 3 + 8 + 13 (in Figure S7); MC3 = MC11 + 12; MC4 = MC10 + 14; MC8 = MC4 + 5. (**B**) FlowSOM trees showing the individual metacluster frequency for each patient. The size of the pie chart corresponds to the frequency relative frequency. (Expression profile: from low (Dark blue) to high (Deep red)).


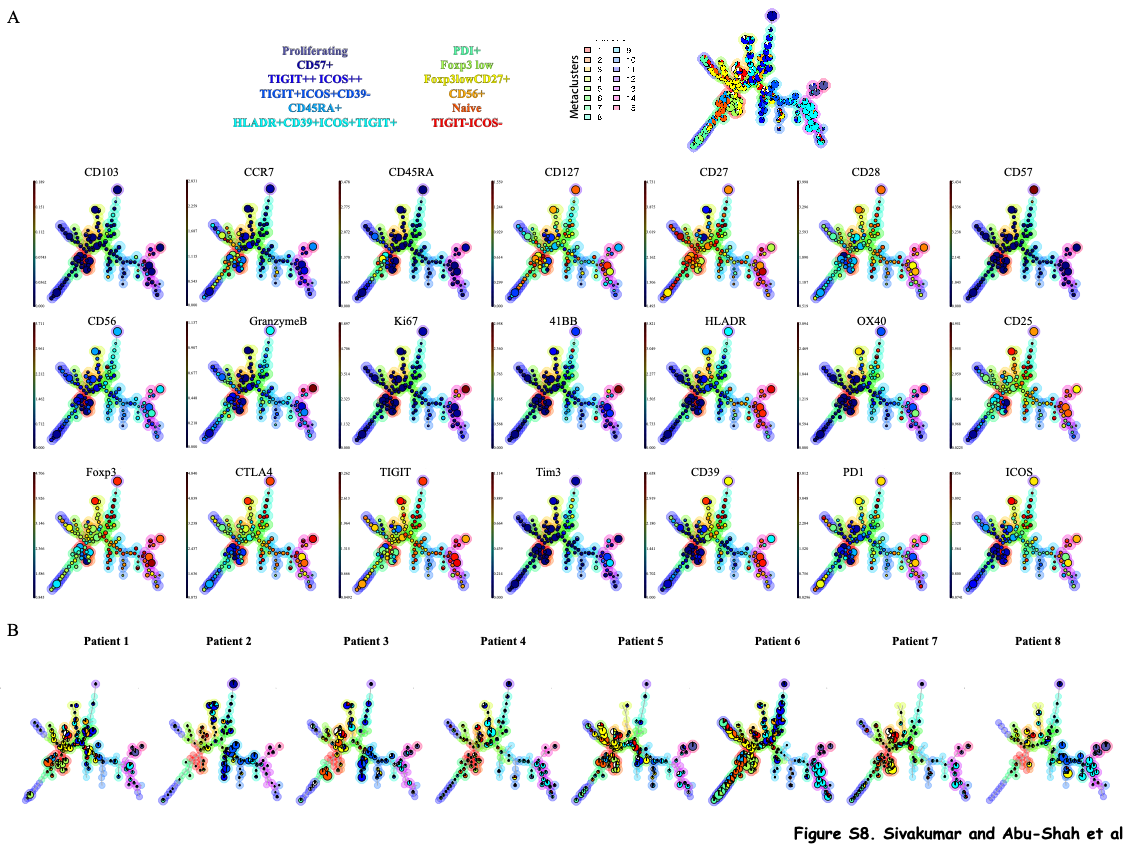


**Figure S8.** Individual patients’ Treg populations. (**A**) Expression of clustering markers on top of a FlowSOM tree showing the relationship between metacluster, for the pooled single-cell data (**A**). Population frequency is presented in the pie chart in each circle along the tree, the background colour represents the metacluster identity. MC1 (in Figure 2C) = MC14 + 15 (in Figure S8); MC3 = MC1 + 2 + 5; MC6 = MC3 + 4 + 6 + 13; MC7 = MC9 + 10. (**B**) FlowSOM trees showing the individual metacluster frequency for each patient. The size of the pie chart corresponds to the frequency relative frequency. (Expression profile: from low (Dark blue) to high (Deep red)).


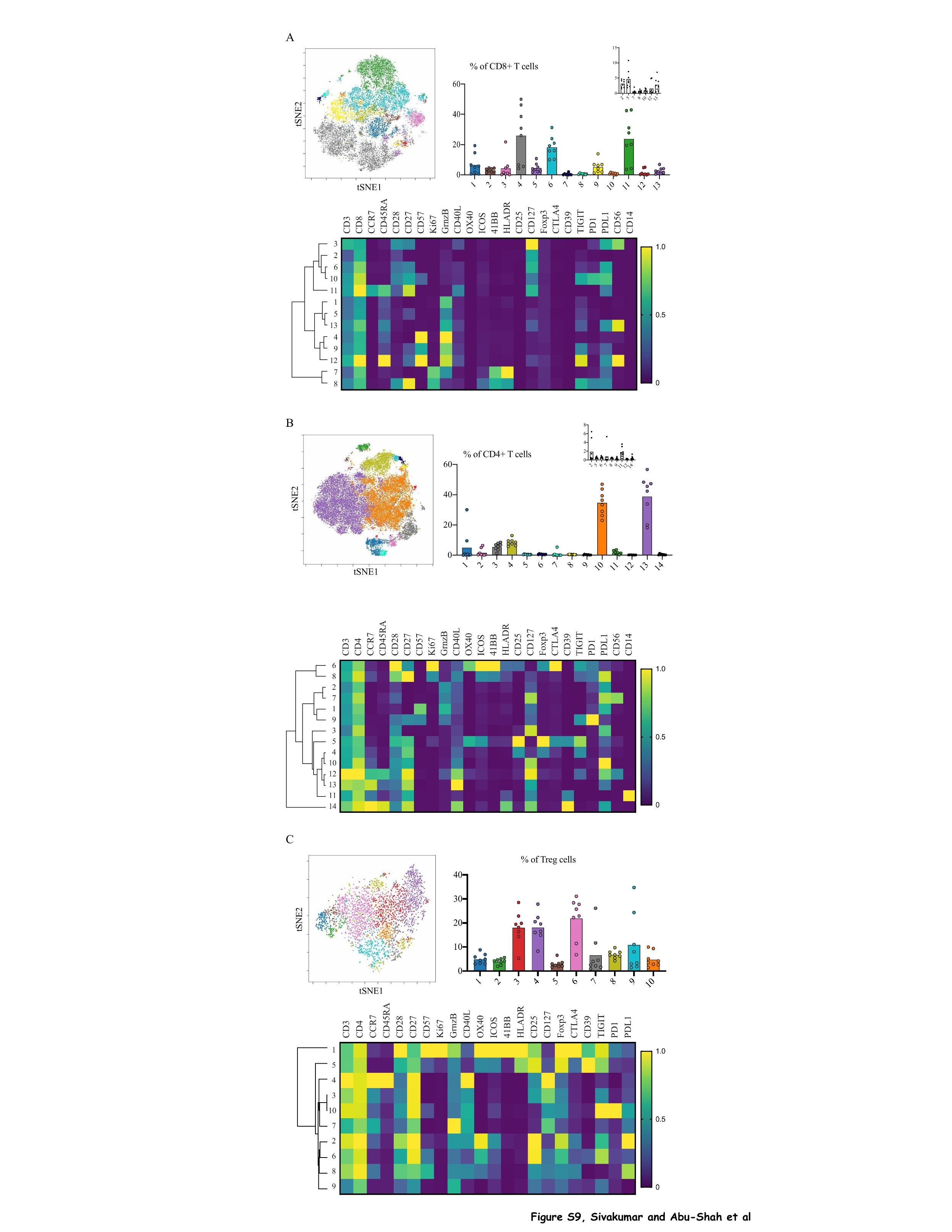


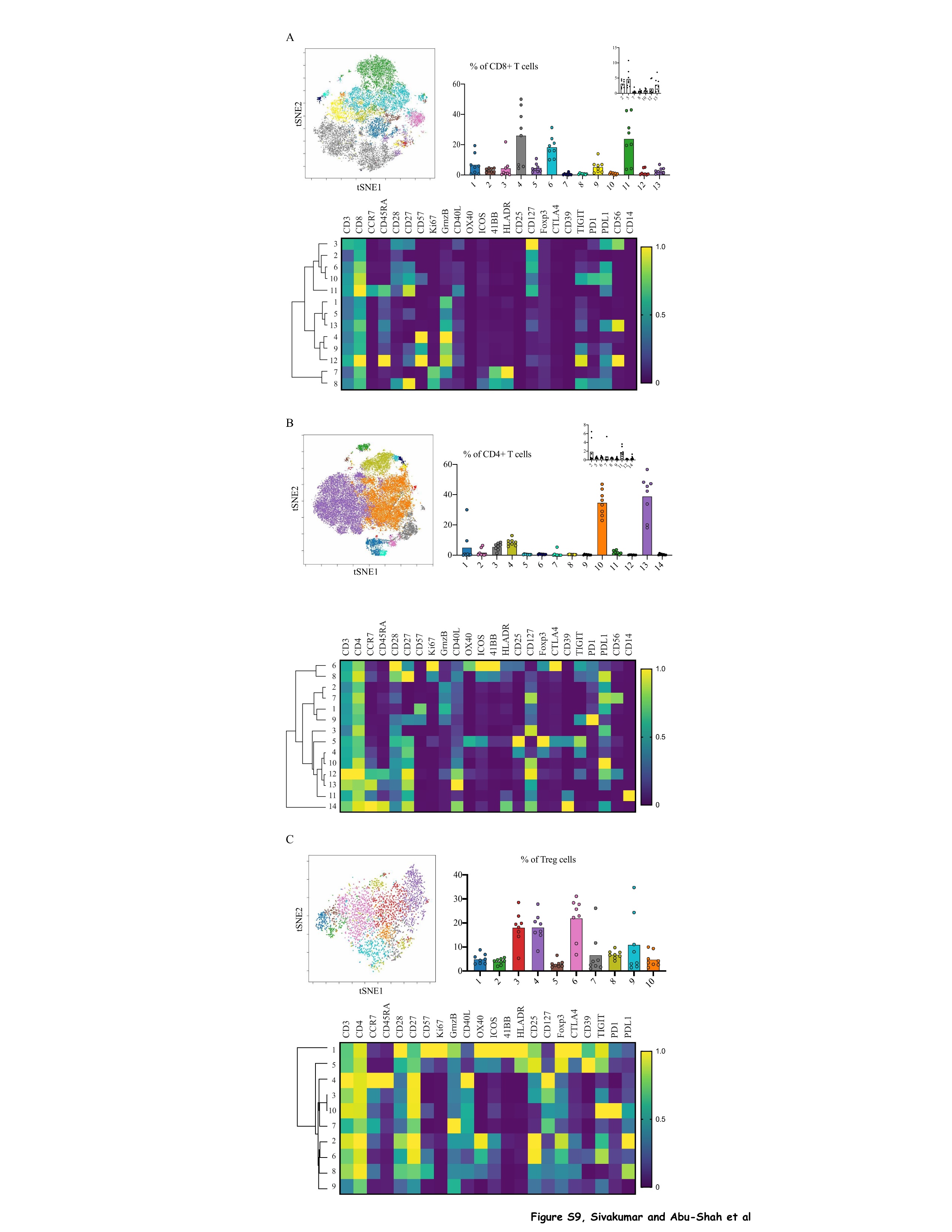


**Figure S9.** Similar T-cell signatures of senescence and suppression to tumours are observed in peripheral blood. (**A**) ~18,270 CD8+ T-cells were pooled and 13 metaclusters identified with FlowSOM and visualised on viSNE plot. Metaclusters’ relative abundance is shown in the bar plot. Inset shows the lower frequency metaclusters. The heatmaps show the expression profile of immune checkpoints in the different metaclusters. Note that the major CD8+ populations are senescent and central memory cells. (**B**) ~38,350 CD4+ T-cells were pooled and 14 metaclusters identified with FlowSOM and visualised on viSNE plot. Metaclusters’ relative abundance is shown in the bar plot. Inset shows the lower frequency metaclusters. The heatmaps show the expression profile of immune checkpoints in the different metaclusters. The major populations are effector and central memory cells, followed by regulatory (Foxp3+) T-cells. Two patients also show a signature of senescent cells in the periphery. (**C**) ~3,600 CD4+ regulatory T-cells were pooled and 10 metaclusters identified with FlowSOM and visualised on viSNE plot. Metaclusters’ relative abundance is shown in the bar plot. Inset shows the lower frequency metaclusters. The heatmaps show the expression profile of immune checkpoints in the different metaclusters. There is a noticeable naive metacluster, and the activated metacluster with similar charactertics to the tumour is observed at lower frequency. Bar plots are medians and each dot represents a patient. Heatmaps were normalised across all T-cell populations per marker. Hierarchal clustering of heatmaps was done in Morpheus.

**Figure S10.** Expression profiles of checkpoints and their ligands. (**A**) viSNE plot of normalised PD-1 expression in CD8+ T-cells. Median expression of PD-1 on different CD8 populations in the tumour and blood. (**B**) PD-L1 median expression on immune, epithelial and stroma cells. (**C**) As in (**A**) for TIGIT. (**D**,**E**) Median expression of TIGIT (**C**) and ICOS (**D**) on different Treg populations in the tumour and blood. (**F**) UMAP showing the main populations of cells in the scRNA sequencing dataset and the expression patter of CD226 (a positive regulator that competes with TIGIT), TIGIT ligands PVR1/2 and ICOS ligand. (**G**) Abundance of cells expressing the ligands in (**F**) between the tumour and a normal pancreas.


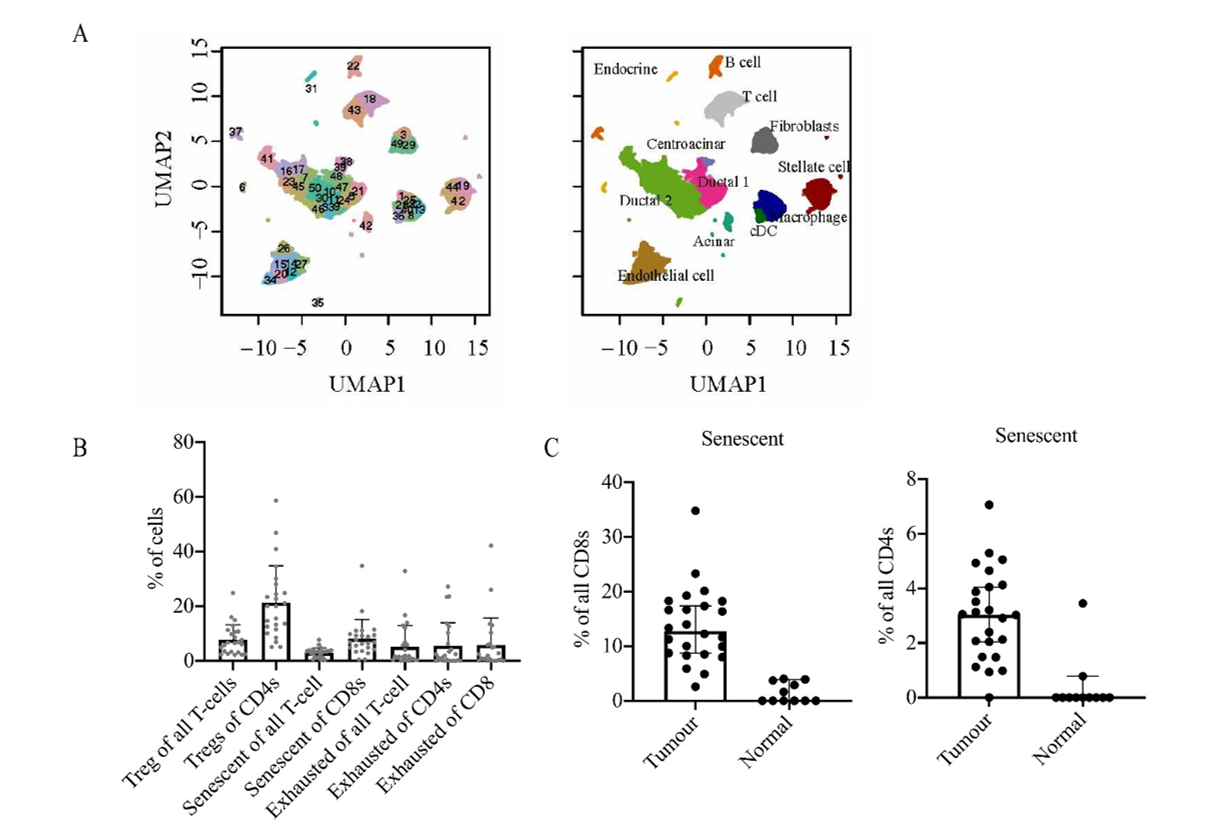


**Figure S11.** Cell frequency validation with scRNA seq. (**A**) UMAP of all cell clusters in scRNA dataset before extracting the T-cell cluster for further analysis in Fig. 3. (**B**) Cell frequencies from Scheme 12. Multiplex cell annotation scheme. (**A**) Cancer region showing H&E. (left), fluorescence image (middle) and cell annotation from HALO (right). Cyanepithelium; magenta- aSMA, green- CD4+, orange- CD8+, yellow- CD3+, purple-Foxp3+, blue- DAPI+. (**B**) As (**A**) for pancreatitis. (**C**) As in (**A**) for normal pancreas. (**D**) Distribution of T-cells across stroma regions with different aSMA densities, where high was defined as the intensity from a blood vessel (CD8+ T-cells, left; CD4+ T-cells middle and Tregs- right).


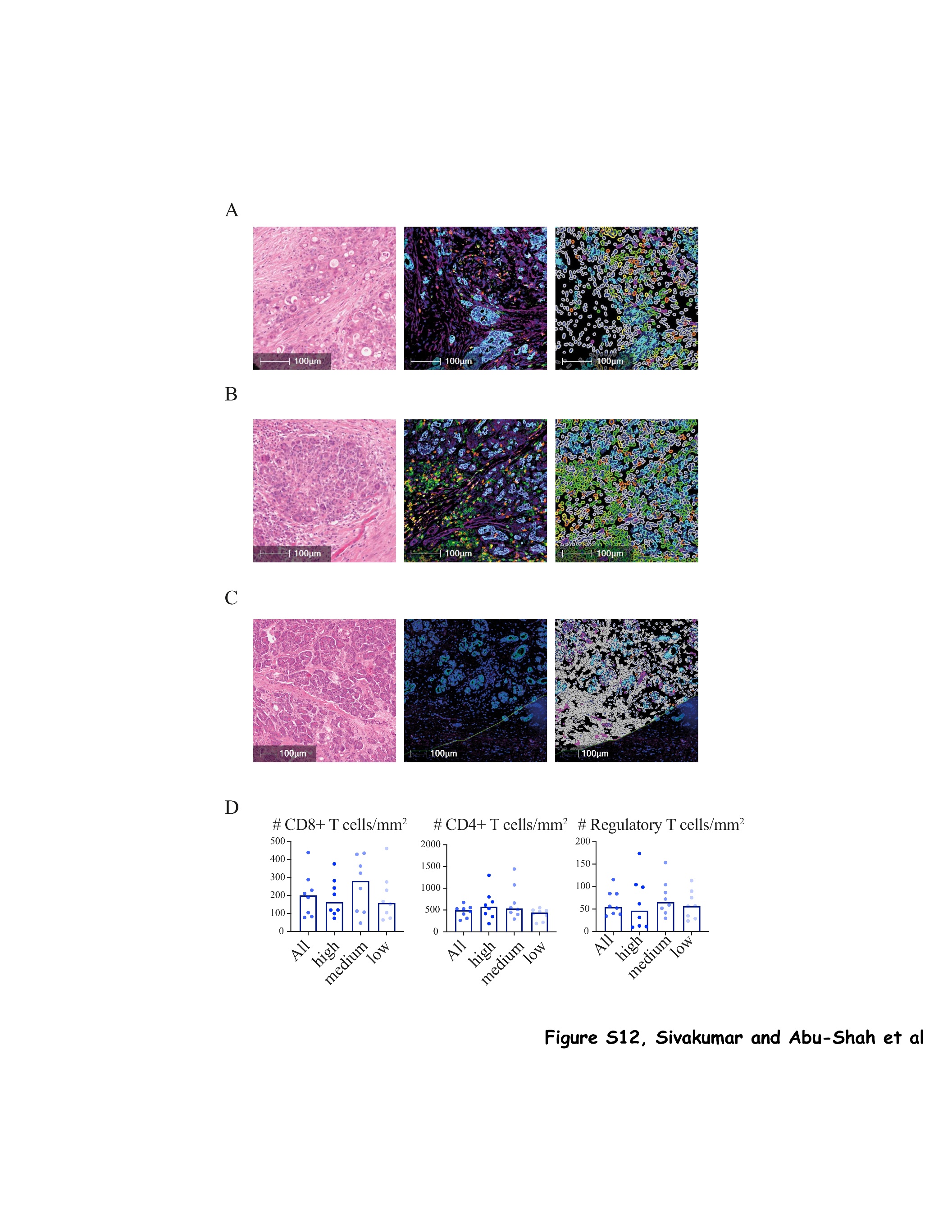


**Figure 12.** Multiplex cell annotation scheme. (**A)** Cancer region showing H&E (left), fluorescence image (middle) and cell annotation from HALO (right). Cyanepithelium; magenta- aSMA, green- CD4+, orange- CD8+, yellow- CD3+, purple- Foxp3+, blue- DAPI+. (**B)** As (**A**) for pancreatitis. (**C**) As in (**A**) for normal pancreas. (**D**) Distribution of T-cells across stroma regions with different aSMA densities, where high was defined as the intensity from a blood vessel (CD8+ T-cells, left; CD4+ T-cells middle and Tregs- right).
